# Progression to invasive cancer after snare polypectomy of intracholecystic papillary neoplasms during gallbladder stone removal by percutaneous transhepatic choledochoscopy: a case report

**DOI:** 10.1186/s12876-020-01547-x

**Published:** 2020-11-30

**Authors:** Chi Hyuk Oh, Seok Ho Dong

**Affiliations:** Division of Gastroenterology and Hepatology, Department of Internal Medicine, Kyung Hee University Hospital, 23, Kyungheedae-ro, Dongdaemun-gu, Seoul, 02447 South Korea

**Keywords:** Intracholecystic papillary neoplasm, Gallbladder, Gallbladder cancer, Acute cholecystitis, Cholecystolithiasis

## Abstract

**Background:**

Intracholecystic papillary neoplasms (ICPNs) of the gallbladder are rare, preinvasive lesions characterized by an intracholecystic papillary growth that may be associated with invasive adenocarcinoma. The natural history of ICPN is unknown. Here, we report a case of ICPN, highlighting its natural course.

**Case presentation:**

A 79-year-old woman presented to the emergency department with perforated cholecystitis. After percutaneous transhepatic gallbladder drainage, due to the presence of surgical risk factors, we opted to perform gallstone removal through percutaneous transhepatic cholangioscopy instead of cholecystectomy. ICPN, which was accidentally detected after the removal of the gallbladder stones, was also endoscopically removed. After 4 years, the patient came back to the hospital with a large gallbladder mass. After cholecystectomy, pathological examination revealed ICPN with invasive adenocarcinoma.

**Conclusion:**

The current case showed endoscopic findings of ICPN and its natural progression, particularly its clinicopathological features and outcomes.

## Background

Intracholecystic papillary neoplasms (ICPNs) of the gallbladder are suspected to be similar to intraductal papillary neoplasms of the bile duct (IPNBs) and intraductal papillary mucinous neoplasms (IPMNs) of the pancreas [1]. ICPNs are rare premalignant lesions characterized by a papillary growth in the gallbladder [2]. Here, we report a unique case wherein progression to invasive ICPN occurred 4 years after the endoscopic resection of ICPN of the gallbladder.

## Case presentation

**Fig. 1:**
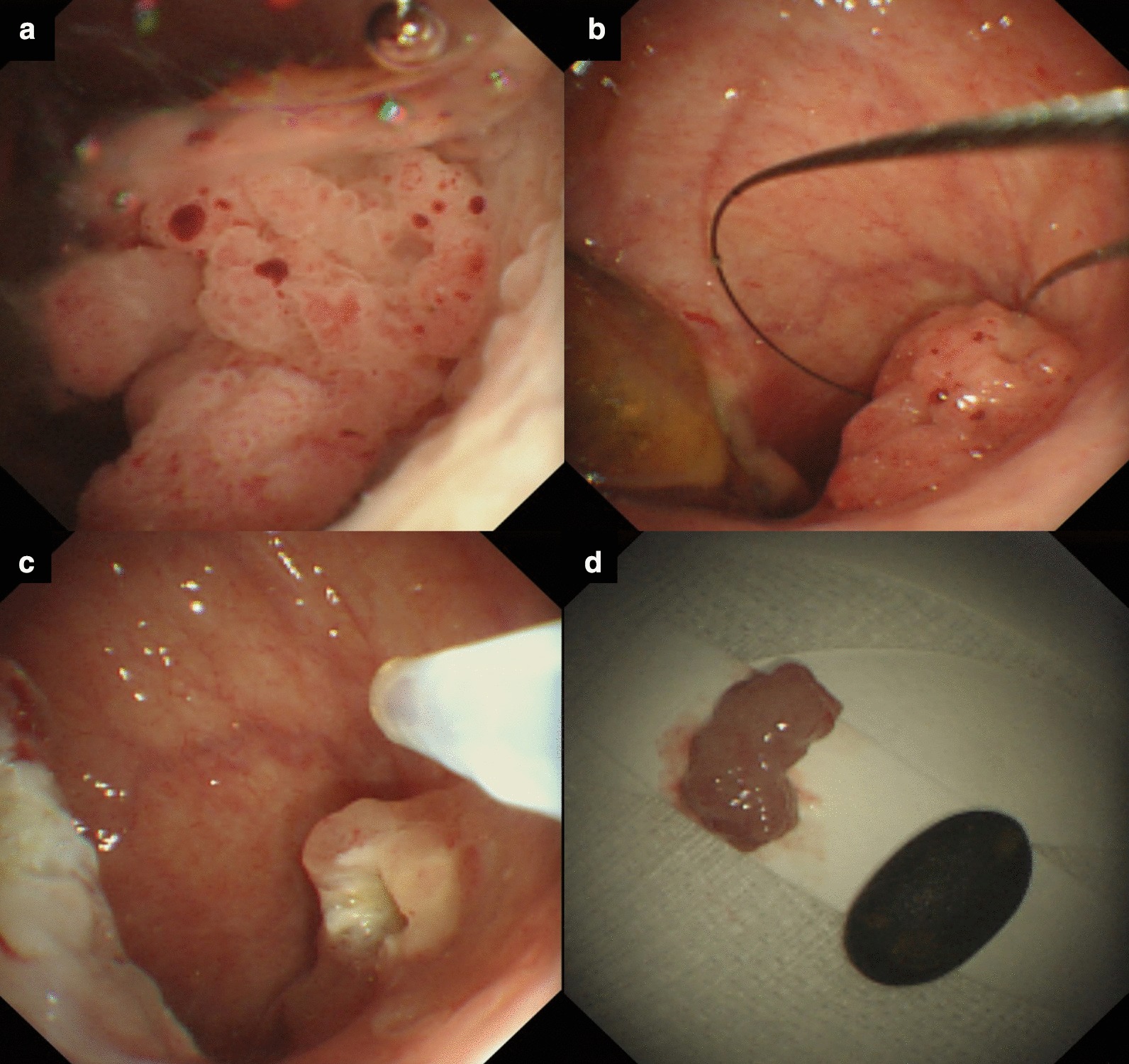
Intracholecystic papillary neoplasm (ICPN) during percutaneous transhepatic choledochoscopy. **a** Papillary tumor within the gallbladder. **b**, **c** Mass removal using snare polypectomy. **d** Resected specimen of ICPN, measuring 1.2 × 0.7 × 0.7 cm, and the removed gallbladder stone

**Fig. 2:**
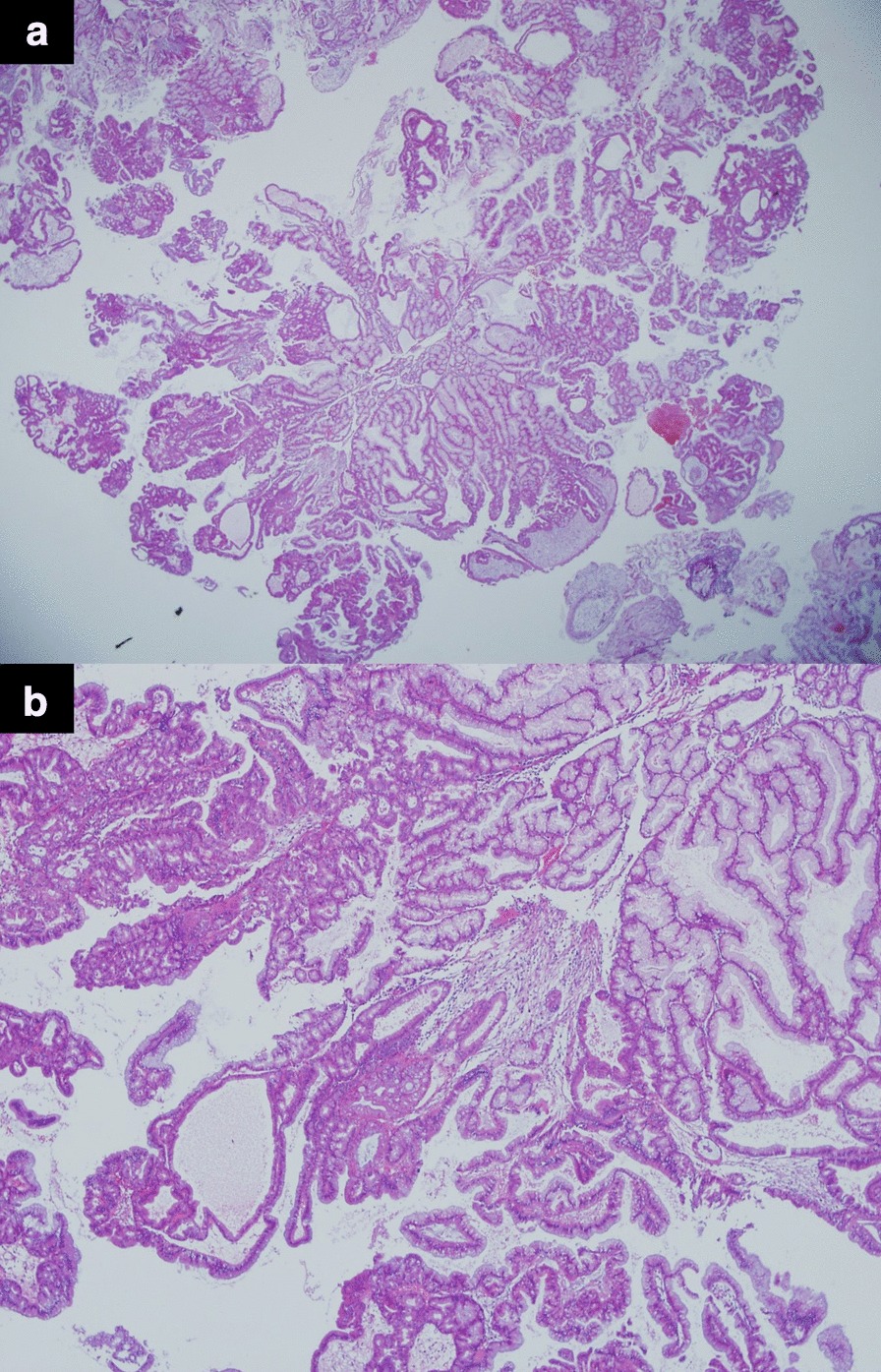
Microscopic findings of the intracholecystic papillary neoplasm (ICPN). **a**, **b** ICPN is characterized by elongated, co-connecting structures and a papillary pattern. It is virtually indistinguishable from the normal glands without cytologic atypia. (**a**: hematoxylin-eosin staining, × 40; **b**: hematoxylin-eosin staining, × 100)

**Fig. 3:**
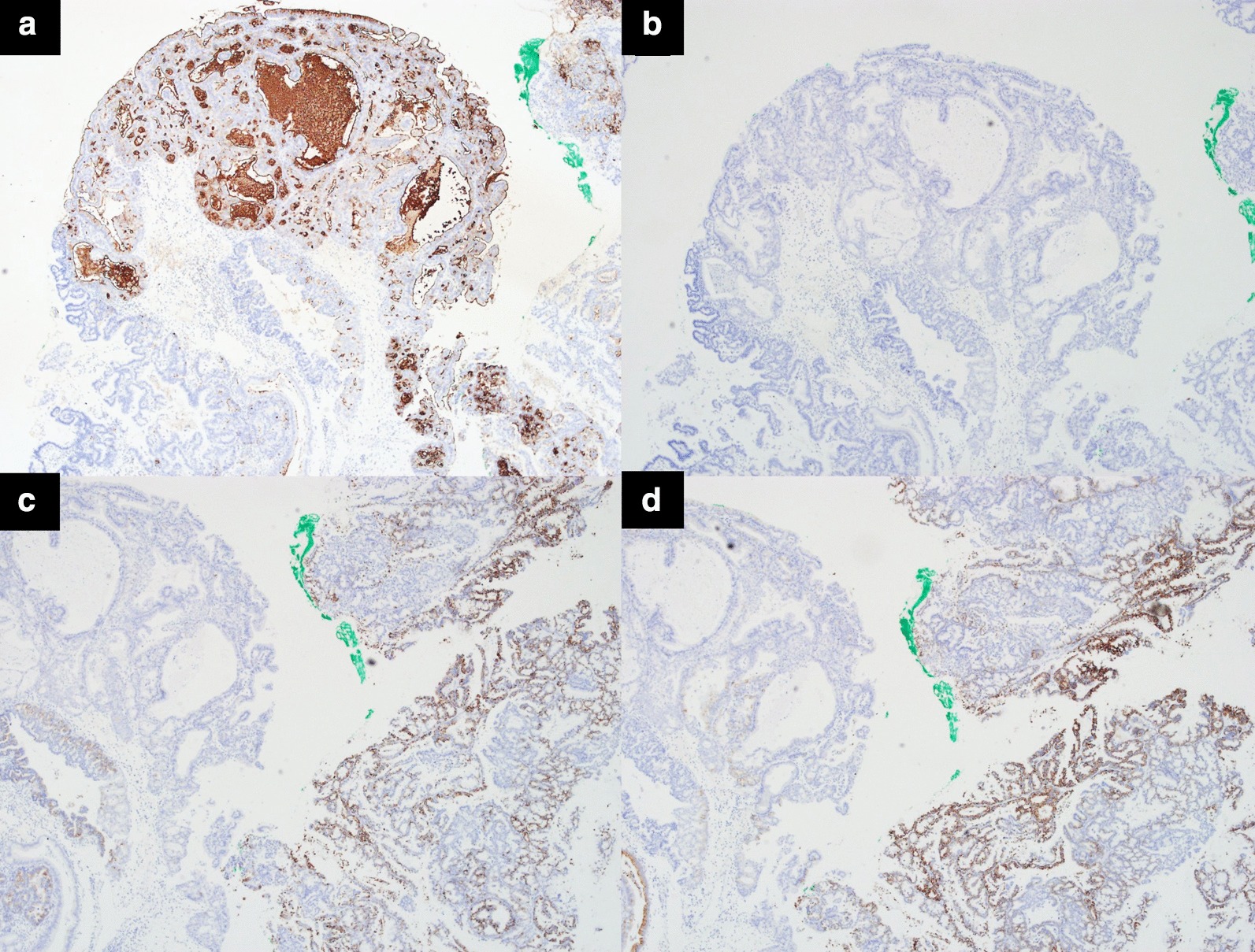
Immunohistochemical stain. **a** MUC1: positive. **b** MUC2: negative. **c** MUC5AC: positive. **d** MUC6: positive

A 79-year-old woman with a recent history of acute myocardial infarction presented with abdominal pain, fever, and altered mental status. The vital signs included the following: blood pressure, 80/60 mmHg; heart rate, 118 beats/min; and body temperature, 39.2 °C. A laboratory analysis showed leukocytosis (white blood cell count, 24,000/μL; seg-neutrophils, 89%) and elevated levels of C-reactive protein (35.30 mg/dL; normal range: < 0.3 mg/dL), total bilirubin (1.6 mg/dL; 0.3–1.2 mg/dL), aspartate aminotransferase (150 U/L; < 50 U/L), alanine aminotransferase (320 U/L; < 50 U/L), alkaline phosphatase (150 U/L; 30–120 U/L), and gamma-glutamyl transpeptidase (350 U/L; 9–64 U/L). Carcinoembryonic antigen and carbohydrate antigen 19–9 were within the normal ranges. Contrast-enhanced computed tomography (CT) showed an approximately 1-cm-sized gallbladder stone, marked thickening of the gallbladder wall, and focal transmural defects with extensive pericholecystic inflammation and fluid collection. Percutaneous transhepatic gallbladder drainage was immediately performed. She was a high-risk patient and not a good candidate for cholecystectomy because of the dual antiplatelet treatment. Moreover, she was strongly averse to undergoing surgery. Therefore, we performed percutaneous transhepatic choledochoscopy (PTCS) for gallbladder stone removal. After stone removal, we found an approximately 1-cm-sized papillary-growing polypoid mass (Fig. 1a). Initially, we attempted to remove the mass using the endoscopic mucosal resection technique. However, mucosal lifting with saline injection was impossible, unlike in the stomach or colon. Therefore, even with the risk of incomplete removal, we removed the mass by performing hot snare polypectomy without submucosal injection (Fig. 1b–d). Histopathological analysis revealed a papillary tumor, measuring 1.2 × 0.7 × 0.7 cm and showing a papillary growth of tumor tissues, which consisted of glandular proliferation (Fig. 2). Immunohistochemical stain showed positive MUC1, MUC5AC, and MUC6 (Fig. 3). It was described as the “gastric type” variant of ICPN. Due to its incomplete removal, cholecystectomy was planned once the patient’s condition improved and she was deemed operable. Unfortunately, the patient was lost to follow-up after discharge.

**Fig. 4:**
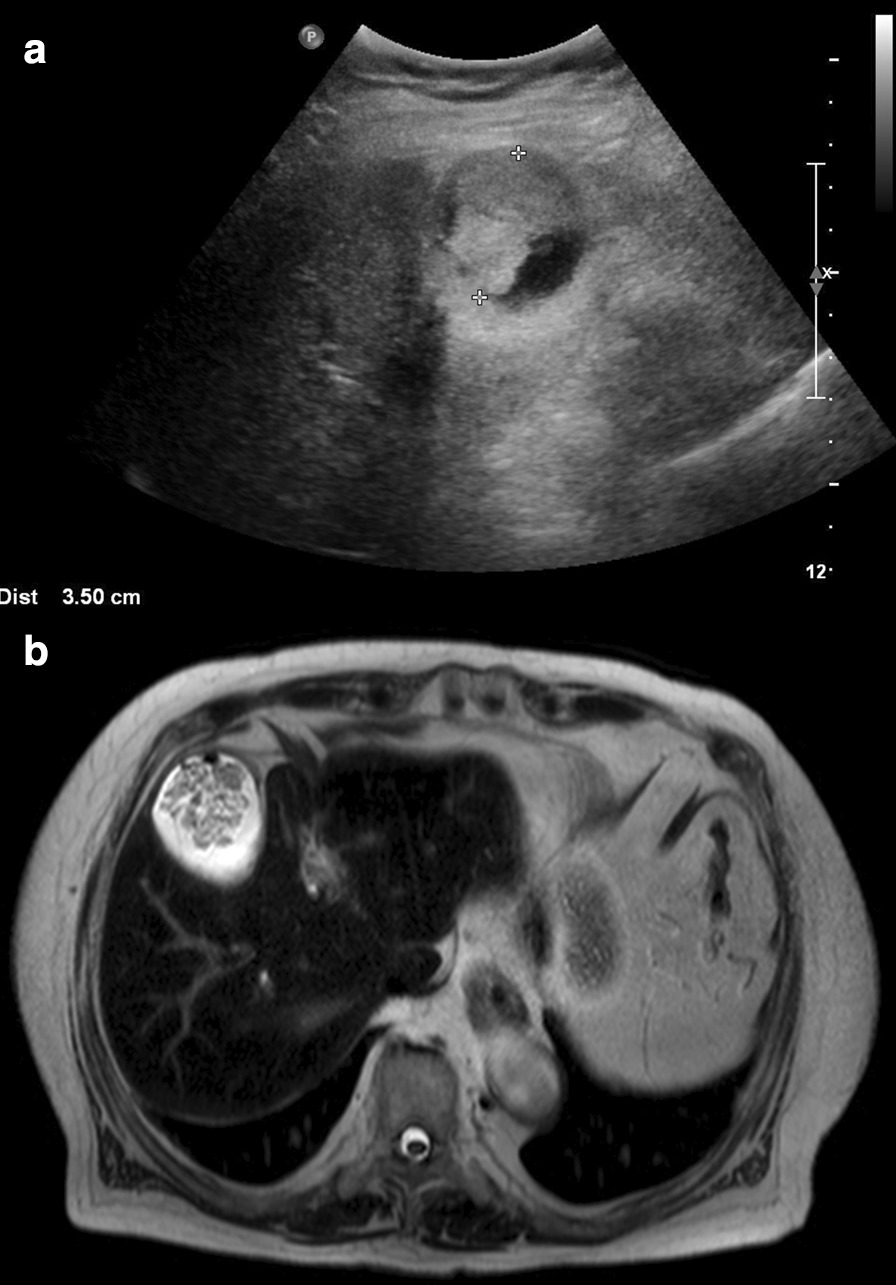
**a** Transabdominal sonography findings. The gallbladder is almost completely filled with a papillary mass. **b** Magnetic resonance imaging findings. T2-weighted magnetic resonance imaging shows a papillary mass within the gallbladder. There is no evidence of gallbladder wall or liver invasion

**Fig. 5:**
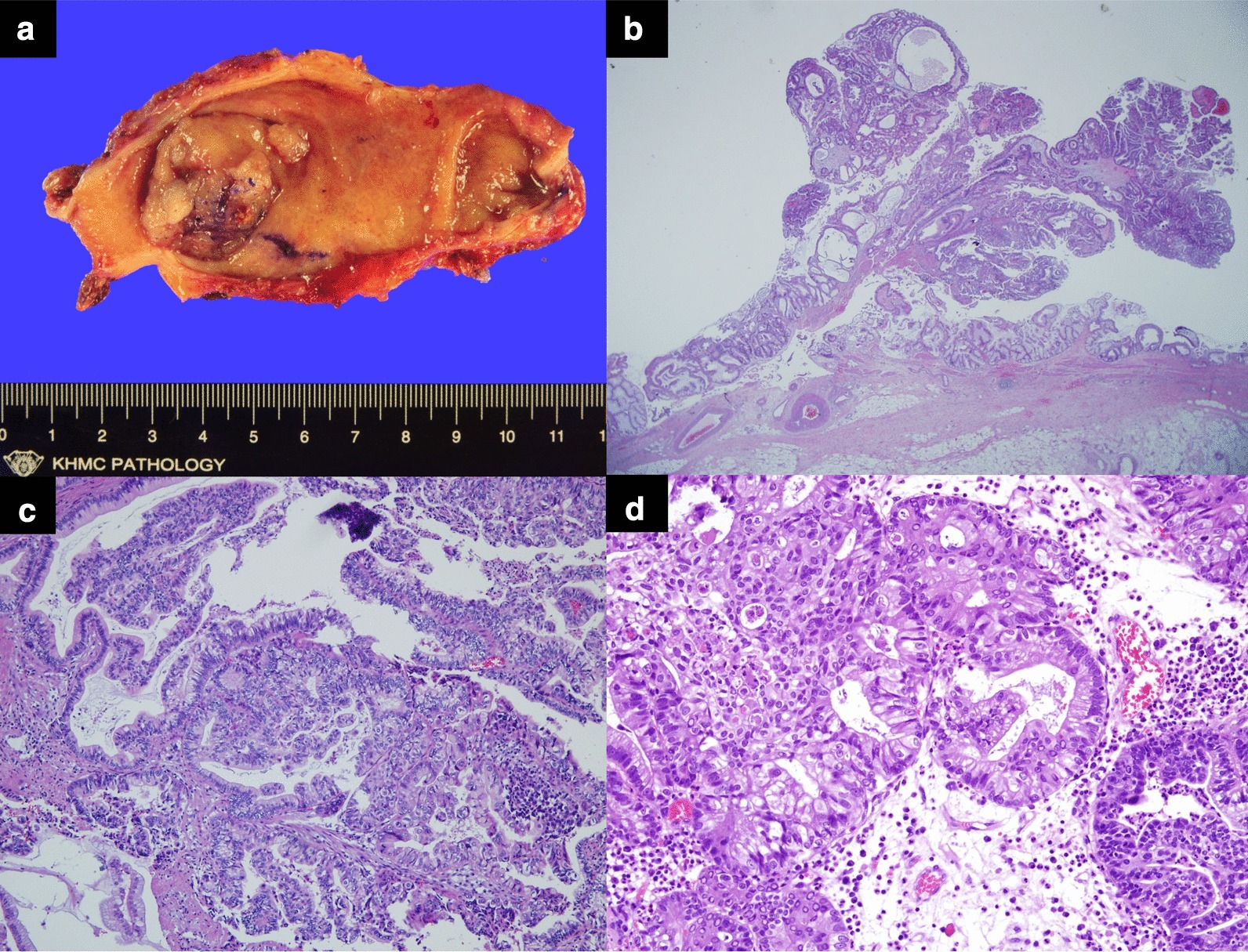
**a** Macroscopic findings of intracholecystic papillary neoplasm (ICPN), measuring 3.0 × 2.0 cm. It is characterized by a polypoid mass protruding into the gallbladder. **b**-**d** Microscopic findings of ICPN. It consists of atypical glandular cells arranged in a papillary architecture. Focal invasion of the lamina propria is observed. (**b**: hematoxylin-eosin staining, × 40; **c**: hematoxylin-eosin staining, × 100; **d**: hematoxylin-eosin staining, × 400)

Approximately 4 years later, the patient came back to the hospital after a mass was detected in the gallbladder on abdominal ultrasonography at another hospital (Fig. 4a). T2-weighted magnetic resonance imaging showed a 3.5-cm-sized papillary mass within the gallbladder (Fig. 4b). The patient’s condition had improved since 4 years before, and dual antiplatelet agents had been discontinued. Therefore, laparoscopic cholecystectomy was performed (Fig. 5a). Microscopically, the tumor consisted of atypical glandular cells arranged in a highly papillary architecture, consistent with adenocarcinoma (Fig. 5b-d). Without adjuvant chemotherapy, the postoperative course was uneventful, and no recurrence was observed at the 3-year follow-up.

## Discussion and conclusions

ICPN is a preinvasive neoplasm of the gallbladder, first mentioned by the World Health Organization in 2010 [3]. Adsay et al. [1] defined ICPN as an exophytic (papillary or polypoid) intramucosal mass measuring > 1 cm and composed of preinvasive neoplastic (dysplastic) cells forming a compact lesion distinct from the neighboring mucosa. They reported that the incidence of ICPN is < 0.5%, and invasive carcinoma is seen in 68 of the 123 (55%) ICPN cases. Despite the possibility of an associated invasive adenocarcinoma, clinical imaging features of ICPN or its natural progression or standardized management strategy have not been reported. Furthermore, approximately 50% of the patients present with no symptoms, and ICPN is incidentally detected during abdominal imaging [4]. Imaging findings of ICPN are similar to those of gallbladder polyps and cancer [5]. Therefore, it is difficult to differentiate them. Some studies have reported on specific imaging findings of ICPN. The typical CT finding is the presence of a > 1-cm enhanced papillary mass in the gallbladder [6]. Additionally, ICPN remarkably exhibits high enhancement from arterial phase to delayed phase. Because ICPN is a mucosal lesion, CT shows no abnormality in the gallbladder wall. Especially in the case of mucin-producing ICPN (intestinal subtype), imaging tests show a distended gallbladder [7].

The prognosis of ICPN is better than that of other invasive cancers of the gallbladder and also depends on the degree of invasion within ICPN [1, 6, 8]. The 5-year survival is 78% for noninvasive ICPN and 60% for invasive ICPN, whereas the overall 5-year survival rate is less than 5% for gallbladder cancer [9]. Thus, even invasive ICPNs have a significantly better prognosis than invasive gallbladder cancers. This can be explained by the polypoid growth of ICPN within the gallbladder, which offers the possibilities of early detection and cholecystectomy. However, prognosis is known to vary depending on the histologic types (pyloric gland, biliary, gastric foveolar, intestinal, and oncocytic subtype) [10]. The pyloric gland subtype is the most common with the lowest risk of invasiveness, whereas the biliary subtype has the highest risk of having invasive adenocarcinoma. Therefore, through immunohistochemical analysis (MUC1, MUC2, MUC5AC, and MUC6), the correct diagnosis and classification become crucial (Table 1). Additionally, microscopic characteristics showed different proportions of invasiveness. In a recent retrospective analysis by Kiruthiga et al. [11], papillary configuration of the ICPN was reported to be significantly associated with an invasive component.

**Table 1:** Immmunohistochemical analysis of ICPN from Hamedani et al. [10]

	Pyloric	Biliary	Gastric Foveolar	Intestinal	Oncocytic
MUC1	+	++	++	+	++
MUC2	–	−/+	–	++	–
MUC5AC	+	+	++	–	++
MUC6	++	+	++	+	+

*ICPN* Intracholecystic papillary neoplasm

Adsay et al. emphasized the importance of long-term follow-up because 3 patients among their 55 patients with noninvasive ICPN died > 5 years after their diagnosis [1]. These patients were reported as having biliary tract cancer, suggesting that they may have suffered from a new primary cancer in the biliary tract. Therefore, after the diagnosis of ICPN, regular follow-up of the remaining biliary tract should be performed.

A few studies and reports have been published on the progression of ICPN. The present case demonstrated the natural progression of ICPN. The ICPN found during the removal of the gallbladder stone was thus not completely removed through PTCS. After 4 years, the remaining lesions had progressed and found to have grown to approximately 3 cm. CT findings were indistinguishable to those of a gallbladder cancer. Cholecystectomy was performed, and the specimen was pathologically confirmed to be ICPN with an associated invasive adenocarcinoma. In a recent case report by Oba et al., a gallbladder adenocarcinoma was found to be localized to the mucosa in association with ICPN through pathological and immunohistochemical analyses [7]. In addition to our case, the synchronic presence of ICPN and invasive adenocarcinoma suggested that the adenocarcinoma was derived from ICPN.

To the best of our knowledge, this is the first case report on the natural disease progression of ICPN to invasive ICPN or adenocarcinoma, both visually and pathologically.

In conclusion, we reported a rare case of the natural progression of ICPN to invasive adenocarcinoma that was successfully treated with laparoscopic cholecystectomy.

## Data Availability

Not applicable.

## Abbreviations

ICPN: Intracholecystic papillary neoplasms
IPNB: Intraductal papillary neoplasms of the bile duct
IPMN: Intraductal papillary mucinous neoplasms
PTCS: Percutaneous transhepatic choledochoscopy
